# A Case of Microscopic Polyangiitis Complicated by Mucormycosis: A Dangerous Balancing Act

**DOI:** 10.7759/cureus.34941

**Published:** 2023-02-13

**Authors:** Ana Valle, Clement Tagoe

**Affiliations:** 1 Division of Internal Medicine, Montefiore Medical Center, Bronx, USA; 2 Division of Rheumatology, Montefiore Medical Center, Bronx, USA

**Keywords:** corticosteroids, rhizopus, mucormycosis, microscopic polyangiitis, anca-associated vasculitis

## Abstract

Microscopic polyangiitis (MPA) is a rare antineutrophil cytoplasmic antibody (ANCA)-associated vasculitis marked by renal involvement, which often leads to rapidly progressive glomerulonephritis. Immunosuppressive treatment is necessary to prevent irreparable organ damage. On the other hand, mucormycosis is a rare and devastating opportunistic fungal infection with a high mortality rate in both immunosuppressed and immunocompetent individuals. It requires a high index of suspicion at the time of diagnosis since any delay in treatment may lead to severe morbidity or death. Here, we present the case of a diabetic patient diagnosed with MPA who received partial induction treatment, subsequently developed mucormycosis, survived, yet required continued immunosuppressive treatment for active MPA while imaging was concerning for a persistent mucormycosis infection. This case highlights the barriers to early mucormycosis detection specific to vasculitis patients, mucormycosis considerations unique to the rheumatologic population, and discusses how to balance immunosuppressive treatment in the setting of a deadly opportunistic infection.

## Introduction

Antineutrophil cytoplasmic antibody (ANCA)-associated vasculitis (AAV) is a group of rare autoimmune diseases defined by small vessel vasculitis associated with the presence of ANCA [1]. Signs and symptoms are variable, given they are manifestations secondary to the inflamed vascular bed and affect the organs reliant on that vessel. The rupture of vessels may cause purpura on the skin or alveolar hemorrhage within the lungs, while vessel occlusion may lead to organ ischemia or infarction. The upper and lower respiratory tracts and kidneys are the organs most commonly and severely affected. Patients usually present with asymmetrical peripheral motor neuropathy and constitutional symptoms, such as fatigue, fever, and arthralgias [1]. Microscopic polyangiitis (MPA) is a type of AAV marked by renal involvement, which often leads to rapidly progressive glomerulonephritis and the presence of myeloperoxidase-ANCA (MPO-ANCA; although proteinase 3 (PR3)-ANCA has also been reported). It has an incidence of 1.5-16 per million person-years, and diagnosis is made with a renal biopsy. Rituximab coupled with systemic, high-dose glucocorticoids is the ideal treatment for active, severe MPA. Cyclophosphamide was previously considered first-line therapy as well, and it remains non-inferior to rituximab when combined with glucocorticoids; however, it is more toxic in comparison to rituximab. Thus, current recommendations only suggest cyclophosphamide as an alternative to rituximab for patients who did not clinically respond to rituximab or were unable to receive it for another reason [2].

Mucormycosis is an opportunistic fungal infection most often described in patients who are immunocompromised due to diabetes mellitus, transplant, or malignancy [3]. With one to two cases per one million people, it is considered a rare disease, although there is some evidence that its global incidence is increasing. Ubiquitous fungal species, such as *Rhizopus* and *Mucor*, are responsible for the infection [4]. These fungi flourish in damp, acidic environments rich in free iron [4,5]. In diabetes mellitus, other mechanisms in addition to free iron, such as the upregulation of fungal proteins and mammalian endothelial receptors, are thought to increase the susceptibility of tissue to fungal penetration [5]. Mucormycosis can disseminate or localize to a specific organ system. Most commonly, it affects rhino-orbital-cerebral areas, leading to acute sinusitis associated with fever, nasal congestion, purulent nasal discharge, and sinus pain. Regardless of location, it causes local destruction, tissue infarction, and necrosis as the hyphae rapidly invade the area [4]. Urgent surgical debridement and amphotericin B are first-line treatments, yet mortality remains high at 46% [3]. Those who survive may be left with disfiguring cosmetic outcomes.

## Case presentation

A 55-year-old female presented to an emergency department in the Bronx, New York, due to bilateral lower extremity edema. She was afebrile and hemodynamically stable. Her past medical conditions included hypertension, hyperlipidemia, and insulin-dependent type 2 diabetes mellitus (hemoglobin A1c (HA1c) 7.7 during this admission). Laboratory data revealed a new creatinine elevation of 1.7 mg/dL and proteinuria of >5 g per day as well as a positive myeloperoxidase antibody of 3 AI determined by enzyme-linked immunoassay. HIV, anti-glomerular basement membrane (GBM), PR3, double-stranded DNA, and Smith antibodies were negative, and complements were within normal limits. She had microscopic hematuria with 4-10 red blood cells/high power field (HPF), and no red blood cell casts were noted. Renal biopsy showed "diffuse crescentic glomerulonephritis, MPO-ANCA associated...no immune complexes seen on electron microscopy." The diagnosis of MPA was made based on these findings consistent with the 2022 American College of Rheumatology/European Alliance of Associations for Rheumatology classification criteria for MPA [6]. She received one dose of cyclophosphamide and began prednisone 60 mg daily before leaving against medical advice.

She continued taking prednisone 60 mg daily at home and did not have any medical follow-up until she returned to the emergency department two months later due to one day of intermittent right facial paresthesia and numbness associated with unilateral blurry vision of the right eye. Stroke imaging was unremarkable, but her glucose level was noted to be elevated to 500 mg/dL. She was admitted for insulin regimen adjustments. Her facial neurological symptoms were attributed to hyperglycemia and AAV. The patient was discharged with a rheumatology appointment and a plan to pursue rituximab to decrease the risk of relapse in the outpatient setting. However, she returned to the emergency department five days later due to a recurrence of right facial numbness, which was now accompanied by left periorbital pain with photophobia, rhinorrhea, and purulent green nasal discharge. She had a temperature of 100.6°F but was hemodynamically stable. Neurological exam confirmed facial numbness in the left maxillary nerve distribution, including hard palate, and ophthalmologic exam was unremarkable.

**Figure 1:**
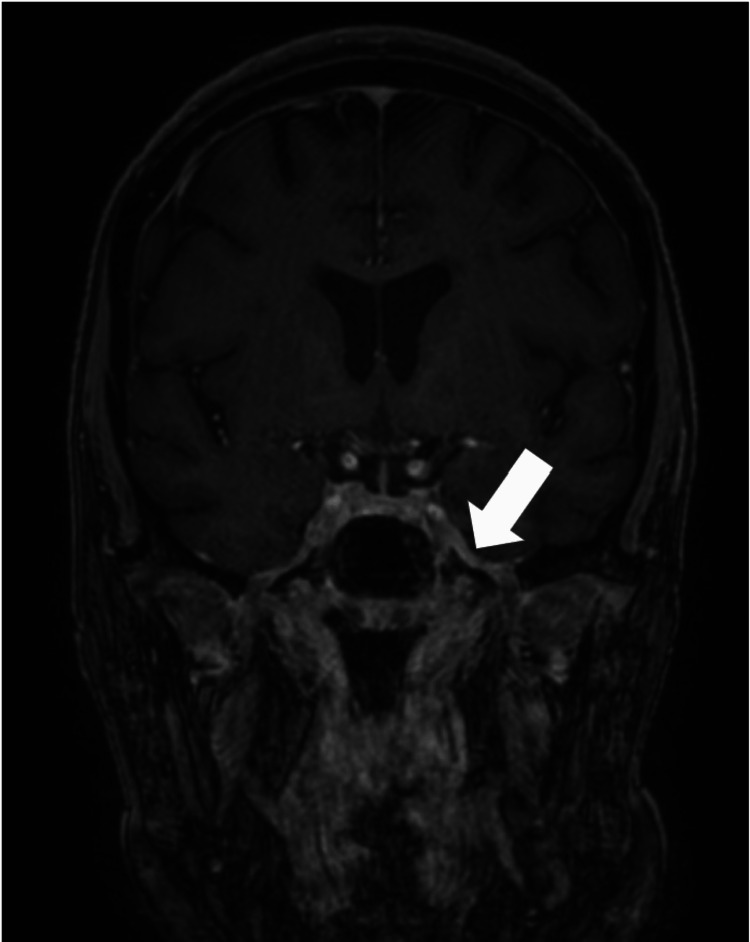
Patient’s first MRI which revealed a mild enlargement and asymmetric enhancement of the left trigeminal nerve maxillary division at the foramen rotundum and mandibular division near its exit through the foramen ovale (arrow).

MRI of the brain and orbits revealed abnormal enhancement in the left masticator space, pterygoid muscles, temporalis muscle, nasal turbinates, and sinuses along with mild, asymmetric enhancement of the maxillary division of the left trigeminal nerve at the foramen rotundum and mandibular division near its exit through the foramen ovale concerning for an invasive fungal species (Figure 1). Direct nasopharyngoscopy evaluation revealed the inferior turbinate appeared dusky. The patient was taken for nasal cavity exploration and debridement the following day. Surgical cultures obtained grew *Rhizopus* and *Serratia marcescens*. Amphotericin B and ceftriaxone were initiated, and prednisone was reduced to 20 mg daily. MRI of the sinuses on postoperative day 2 revealed increased enhancement, so the patient returned to the operating room for further debridement of necrotic tissue. A repeat postoperative MRI on postoperative day 4 once again revealed continued abnormal enhancement that was worse in comparison with previous imaging, and the patient returned to the operating room for additional debridement. The patient completed a two-week course of amphotericin B followed by posaconazole for six months, atovaquone for *Pneumocystis jirovecii* pneumonia prophylaxis, and continued prednisone 20 mg daily

**Figure 2:**
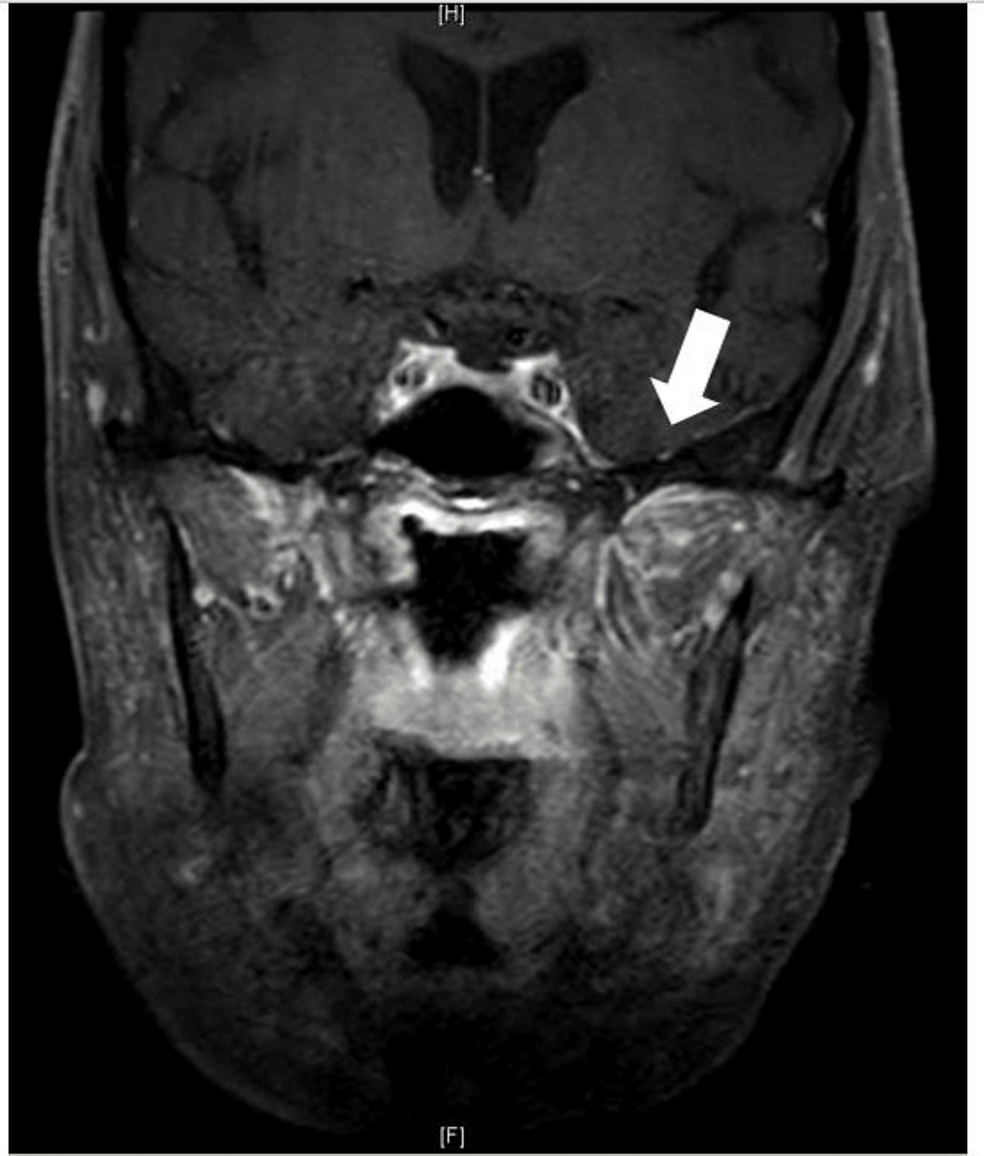
Patient’s last MRI which revealed a persistent abnormal signal and enhancement in the left pterygomaxillary fissure and left foramen rotundum although mild dural enhancement along the left medial middle cranial fossa had decreased (arrow).

An MRI of the sinuses and orbits two months after debridement revealed decreased but persistent abnormal enhancement in the left retromaxillary fat and masticator space, left periorbital soft tissues and orbit, left pterygomaxillary fissure, left foramen rotundum, and in the dura along the left medial middle cranial fossa, left inferior anterior cranial fossa, and right inferior frontal lobe (Figure 2). Through shared decision-making, it was decided to proceed with decreasing her prednisone dose to 5 mg daily and closely monitor without further debridement.

Outcome and follow-up

Since her debridement, the patient has had a persistent, unchanged loss of sensation in the distribution of the left maxillary nerve. Over the next year, she remained on prednisone 5 mg daily. Her kidney function continued to deteriorate, and 15 months after her initial presentation of MPA, she transitioned to end-stage renal disease and initiated hemodialysis. At the time of this paper, the patient has not had any recurrence of mucormycosis or MPA in other organ systems.

## Discussion

This is the case of a diabetic patient who was diagnosed with MPA that required high-dose glucocorticoids and subsequently developed mucormycosis. The development of mucormycosis in a diabetic is well documented [3,5,7,8]. However, one must also speculate whether her rheumatic condition or its treatment contributed to the development of mucormycosis. Furthermore, the non-fatal and relatively chronic disease course of this patient’s mucormycosis requires examination and begs the question of how to balance a lethal opportunistic infection with AAV treatment to preserve organ function.

The use of biologics has vastly improved AAV outcomes; greater than 90% of patients now achieve remission in what was once a deadly disease [9]. However, this requires tolerance of immunosuppressive regimens, which almost always include high-dose corticosteroids, which may lead to infection and may be limited by renal impairment. In fact, infection is now the leading cause of death within the first year of AAV diagnosis rather than active disease [9]. Our case is an example of how treatment can be repeatedly delayed due to active infection. After stabilization of the fungal disease, MRI was concerned for persistent infection in the cranium base, as seen in Figure 2. Rituximab, azathioprine, or another potent biologic could not be considered when the risk of mucormycosis reemergence was possible. Intravenous immune globulin (IVIG) had been discussed as an alternative to biologics, but our patient faced significant financial and social barriers obtaining this medication. One must also recall that its use in AAV is not discussed in the most recent treatment guidelines [2]. At the time of this case, there were no alternatives available to steroids. However, avacopan, a steroid-sparing complement C5a receptor inhibitor, has recently received approval [10]. Its performance in comparison with steroids was non-inferior, and it had a decreased risk of infection in comparison to glucocorticoids [11].

Despite advancements in AAV treatment, mucormycosis remains a lethal infection, with the majority of cases diagnosed postmortem. A case described a diabetic AAV patient who expired shortly after presenting with a frontal headache, imaging consistent with sinusitis, and a cerebrovascular event attributed to an AAV flare. The diagnosis of mucormycosis was only established at autopsy [12]. Our patient initially presented with right facial paresthesia and numbness associated with unilateral blurry vision of the right eye. Her symptoms were initially attributed to cranial nerve palsy secondary to severe hyperglycemia and an AAV flare. Only when she presented again with more classic mucormycosis symptoms, such as purulent green nasal discharge, did she receive an accurate diagnosis. This highlights the difficulty of distinguishing vasculitis from infections with similar presentations.

One case of mucormycosis in a patient who presented with diabetic ketoacidosis after cyclophosphamide induction and three months of steroid treatment for idiopathic rapidly progressive glomerulonephritis has been described. However, this patient’s renal disease was near remission, so prednisolone was discontinued soon after [6]. In comparison, our patient still had active, rapidly progressive glomerulonephritis with worsening renal function. This is compounded by amphotericin B’s nephrotoxicity, even when a lipid-based formulation is used. Studies have questioned whether there is a role for antifungal prophylaxis in immunocompromised patients, such as the case we have described here. However, there is little support for this due to increasing antifungal resistance and breakthrough mucormycosis infections [3].

While our patient’s diabetes may have potentiated the development of mucormycosis, the role of her autoimmune disease must be emphasized. It was due to MPA that she began chronic, high-dose corticosteroids, which is a risk factor for mucormycosis independent of diabetes [13]. In a systemic review of mucormycosis in rheumatic diseases, Royer et al. found that the majority of patients (14/22) lacked a history of diabetes, yet all of them were exposed to corticosteroids [14]. This hints that patients with rheumatic diseases or the treatment of these diseases may also modulate their risk of mucormycosis. In addition, one can speculate whether the local nasopharyngeal destruction caused by MPA may have created a nidus for the rhino-cerebral mucormycosis infection. Penetrating trauma has been known to predispose patients to mucormycosis. However, this is far more frequent in cutaneous forms of mucormycosis [15]. Furthermore, due to our patient’s MPA, there was a delay in the diagnosis, given the similarities in presentation between AAV and mucormycosis. This raises the concern that mucormycosis infections in AAV are underreported and likely undertreated, despite deadly outcomes. Another review of mucormycosis in patients with autoimmune conditions reported a higher mortality rate of 58% in this population, which may be due to delays in diagnosis and balancing immunosuppressive medications, both of which we described in our case [14]. We recommend a high index of suspicion of mucormycosis in immunosuppressed patients. Additional reporting of these cases is necessary to develop alternative biologic and steroid-sparing, evidence-based treatment protocols that successfully achieve AAV remission without organ failure during an opportunistic infection.

## Conclusions

Here, we presented the case of a patient with MPA who developed mucormycosis and survived despite delays in both the diagnosis of the deadly opportunistic infection and MPA treatment due to the active infection. Despite the patient’s history of diabetes, this case highlights the barriers to early mucormycosis detection specific to vasculitis patients given the similar presentations between MPA and rhino-cerebral mucormycosis and reviews treatment alternatives, including avacopan and intravenous immune globulin therapy. It reinforces mucormycosis risk factors, such as steroid exposure, which are independent risk factors apart from diabetes, and highlights the need for more investigation regarding how destructive autoimmune processes themselves potentiate the risk of mucormycosis in addition to autoimmune disease treatments. As the global population of immunocompromised individuals increases, this case report is pertinent to increase discussions about balancing the diagnosis and treatment of autoimmune conditions with lethal opportunistic infections.
